# Supplementary material for: DIVE: a reference-free statistical approach to diversity-generating and mobile genetic element discovery
Source: Genome Biol. 2023 Oct 20;24:240. doi: 10.1186/s13059-023-03038-0 (PMC10589994; doi:10.1186/s13059-023-03038-0)
Supplement: Supplementary file 4 — Additional file 4. Peer review history. [file 13059_2023_3038_MOESM4_ESM.docx]

**Review history**

**Note:**

This manuscript was previously peer reviewed by another journal, and subsequently submitted to Genome Biology in a revised version with comments responding to reviewers’ criticism. The two reviewers below are those who reviewed the work previously.

**Authors’ response to reviewers at previous journal**

## Reviewer 1

### 1. The possibility to discover new variable regions and MGE using in silico method is very useful. However, it seems that the last part used by the authors to ﬁnd those (assembly using the seeds as anchors and domain annotation) is not present in the tool. Can the authors explain the process and why it is not incorporated inside the BioDIVE tool? Because from our current understanding and use of the tool, we were not able to reproduce the types of analysis proposed in the paper on our own datasets, probably due to lack of clarity more than a method problem.

The reviewer is correct that the assembly and protein homology analysis steps are not part of the Python package biodive. This is because this analysis can be done by many tools that are already publicly available and different researchers might have different preferences. In our case, as it is outlined in the Methods section, we use SSAKE (v4.0) for seed-based assembly and we use HMMER (v3.3.2) for protein domain homology analysis.

For each sample, putative termini are identified by picking representative anchors (RAs) that show significant variability in one direction. This was mentioned in Fig. 3, but we have made it explicit in the text. The rationale behind this choice is that we expect termini to show diversity only on the insertion end (in contrast to DGMs such as CRISPR repeats). The putative termini are then used as seeds in the assembly step. Given the lack of consensus in the direction of variability, the contigs tend to grow inwards of the element (provided the sequence of the element is conserved enough) until it reaches the other end of the element, where there is a lack of sequence consensus. This is done through an iterative process as described in the Methods section. The resulting contigs can then be filtered based on the presence or absence of known protein domains. In our case, we use transposon-associated domains to filter the contigs. However, one could look for domains associated with other MGEs of interest (phages, ICE, etc.). Following these steps, we get contigs for each one of the 75 isolates. However, many of these contigs could overlap with each other and, in turn, we use CD-HIT-EST to consolidate all the contigs into a list of contig clusters.

The reviewer, however, brings up an excellent point regarding the lack of clarity regarding the level at which the assembly and homology steps are done. We have clarified that this analysis is done on a per-sample basis in the Methods section *Anchor extension and protein domain homology****.*** In addition, and for the sake of reproducibility, we have created a separate Github [repository](https://github.com/jordiabante/DIVEpaper) to include the source code we used both for assembly and protein domain homology analysis.

### 2. Maybe the authors should be more cautious when they say that “Building on DIVE, we develop a reference-free framework for de novo discovery of mobile genetic elements”. One would expect that discovery of mobile genetic elements includes prophage, ICE and any related elements. In fact it seems that it can detect transposons (any size of Tn?) which requires additional steps not included in the software. Could the authors comment on the possibility to detect other types of MGEs? Otherwise, DIVE appears to be able to be good at ﬁnding variable but meaningful variable region such as CRISPR repeats and hotspots regions. This is valuable if the authors shows that it performs better than MGEﬁnder for this task.

DIVE will detect the termini of MGEs for which (i) there are multiple copies in the sequenced genome, or (ii) it’s inserted in different loci across the sequenced DNA sequences. The two scenarios are considered in our simulations (Main text, Supplementary Information - Simulations, Figures S2-S6), where the performance of DIVE is benchmarked against MGEfinder, the state of the art, showing that DIVE consistently outperforms MGEfinder throughout different coverages, MGE activity levels, and within-sample genetic variability. In fact, we use the same type of simulations that MGEfinder used in their benchmark, also using simulations and assessing the performance of the tool based on the detection performance of MGE termini.

However, DIVE does not do *de novo* MGE discovery *per se*. That is why we preface the quotes sentence with *“Building on DIVE, …”*. As outlined in our response to the reviewer’s point, putative MGE termini can be obtained from DIVE’s output by keeping representative anchors that show significance in a single direction (see above for more detail). This can then be followed by a seed-based assembly where the anchors are extended to reconstruct the inner part of the MGE (e.g., SSAKE). Subsequently, protein domain homology can be used to pull out the contigs containing domains that are associated with a specific MGE of interest (e.g., HHMER). This is the framework we propose for reference-free *de novo* discovery of MGE. We show the validity and potential of this framework by reconstructing *de novo* the sequence of IS6110, among nine other transposon sequences, in *M. tuberculosis* without using a reference genome. As explained in the manuscript, to the best of our knowledge, this is the first time this has been done.

To reflect this clearly in the text, we have clarified (i) what the proposed framework consists of, and (ii) that the transposon *de novo* detection example is just an application of the proposed framework.

### 3. According to the method in the manuscript, the dive analysis seems to be done using reads coming from different genomes to detect anchors linked to MGE. However, in the biodive GitHub, the readme talks about a “single sample analysis”. Can the manuscript or the readme be clearer about which input is needed to run the analysis? It is also necessary to indicate the boundaries of which the method is useful; typically a benchmark of how good the method is given number of reads and number of different strains.

We realize the text might not have been clear about this and we have clarified that DIVE takes as input a single FASTQ file in the main text. As the reviewer pointed out, this is already clear in the GitHub repository where we only discuss “single sample analysis”.

We also agree that a benchmark can be very useful and that is why we have performed extensive simulations in two different scenarios (Main text, Supplementary Information - Simulations, Figures S2-S6). First, we look at a setting where an MGE is found in a given set of loci and we vary the copy number of the element, the coverage of the sample, and the genetic divergence within the sample. Second, we consider a setting where the element is found in different loci across the sample and we vary the activity level of the element, the coverage, and the genetic divergence within the sample. This is also the setting where we compare the performance of DIVE to that of MGEfinder in detecting MGE termini, showing that DIVE outperforms MGEfinder in almost all considered scenarios. These simulations give a good idea of the boundaries and limitations of DIVE.

Given the limited space, these simulations are not discussed in great length in the main text. However, the main points are made in the paragraph that starts with *“Next, we performed a set of simulations (...)”.*

### 4. The rational with the current simulation’s procedure is not extremely clear, and in general the choice of some parameters is hard to comprehend. Why simulating genome of 100kb long and not a realistic value like 4Mb? Why inserting mutation at random and not using proper simulation software which will produce realistic patterns of mutation distribution (e.g. msprime or SLiM) ? Why not using pseudogenomes (maintaining tri or di nucleotide composition of known genomes, with e.g. varying GC content to assess this).

Since each configuration needs to be simulated a thousand times to be able to produce an accurate receiver operating curve (ROC), and we have over 100 different settings that we are considering, we had to settle on a 100 kb genome as a compromise due to computational and time limitations. However, we believe that the presented simulations are realistic enough to show the performance of DIVE, understand the limitations of the proposed approach, and compare it to MGEfinder. Indeed, any tool that is required to work with 4 Mb genomes should work just fine with a 100 kb genome.

We emphasize that the presented simulations include varying (i) the copy number of the MGE, (ii) the activity level of the MGE, (iii) the sequencing depth/coverage of the sample, and (iv) the genetic divergence within the sample through mutations and indels. Replacing these simulations with software that performs more realistic mutation patterns will have minimal impact on the results.

### 5. Why taking a FDR of 10% in the BH correction when 5% is usually considered or even 1%. 10% of false positives seems high?

We respectfully disagree with the reviewer on this point - in our experience, a 10% cut-off for Benjamini Hochberg-corrected p-values is not uncommon.

However, It is true that within the *de novo* discovery setting increasing the FDR threshold will require more computational resources during the assembly step as the number of seeds used per sample increases. However, we believe that the extra cost during the assembly step due to a lenient FDR threshold is worth it since at the same time this is allowing DIVE to be more sensitive in detecting true MGE termini. Furthermore, we expect most of the false positives to go away upon filtering the resulting contigs, and this is indeed what we see in our *de novo* MGE discovery in *M. tuberculosis*, where all the contigs after filtering appear to be true positives.

Most importantly, however, DIVE does produce BH-corrected p-values which can then be used by the user as desired. This is in contrast to MGEfinder, which to the best of our knowledge, does not produce any quantity that allows one to compute a ROC curve. We strongly believe that is the case provided they did not provide these curves in their paper either.

### 6. The clustering of targets sequences relies on the Jaccard similarity measure between 7-mers of two 27-mers (the two targets to be compared). Why clustering with a score of 0.2 or more which would represent 4 out 20 7-mer in common between the two 27mers (if I understood well). It seems like a light threshold.

The reviewer brings up a bring point that, due to space limitations, we could not discuss in the text. We have added a brief discussion about this to the *Methods* section *Target sequence hyper-variability*.

An interesting property of the hypothesis testing framework proposed (see *Target sequence hyper-variability* in *Methods*) is that it adapts to the Jaccard similarity (JS) threshold set by the user. The JS threshold determines the propensity of creating new target clusters, and the rate at which these are created is the test statistic used to compute a p-value. The null distribution of this test statistic is estimated from the dataset and thus is created for the specific JS threshold chosen by the user. This threshold needs to be small enough so that whenever there is true diversity new target clusters are formed. Otherwise, the test statistic would render useless since it would not generate any differences between true positives and negatives. At the same time, however, having a very high JS threshold would result in very large target dictionaries since for every new sequence observed with a call error a new target cluster would be created. Even though the hypothesis testing framework could handle this situation, this would require a lot of RAM since the target dictionary of every single anchor would grow substantially.

In our experience, this threshold achieves a good compromise between these two considerations and, as our simulations and real data results show, the threshold allows for large numbers of true positives, while properly controlling the type I error.

### 7. How were determined the Nmin and Nmax, is there any rational?

We determined N_min_ and N_max_ considering a few factors. Note that this choice does not after the validity of the statistical test since DIVE controls for the anchor coverage.

An important factor in determining the minimum sample size *N_min_* is that the statistical test does not have as much statistical power for small N. In general, the power of any statistical test grows with the sample size and DIVE is no exception to this rule. Thus, performing a statistical test for an anchor with a small N will rarely yield any positives. However, the

p-value produced will have to be accounted for during multiple hypothesis testing correction. Thus, not only the test will likely lack power, but it’ll also reduce the overall power of the method. As a result, we decided to test only the anchors for which we clearly have statistical power. Thus, we set *N_min_=25* for this precise reason.

An important factor in determining the maximum sample size *N_max_* is the computational resources available. Similarly to the JS threshold, increasing the maximum sample size will increase the target dictionary size for all anchors, requiring more RAM to run DIVE. Since the test is based on the rate at which new target clusters are observed, and not the total number of target clusters created alone, the power of the test is already rather large with a sample size of 75. Thus, we set *N_max_=75* for this reason.

In our experience, we found that these are good values for our application. However, we also note that these parameters can be modified by the user depending on the coverage and computational resources available.

### 8. With regards to how DIVE compare with other software, it’s not clear why the fact that MGEFinder does not use p-value prevents the production of ROC curve (we are not users of MGEﬁnder so we might miss something here) but this should be made clearer. Also, DIVE and MGEﬁnder identify different number of TE in N gonorrhoeae. Other comparisons could be made with other programs detecting more speciﬁc MGE (such as virsorter).

To the best of our knowledge, MGEfinder does not report any quantity that can be used to obtain a ROC curve (see *Comparison with MGEfinder* in *Supplementary Information*). In fact, in the original publication (Durrant, 2020) there are no ROC curves either, only sensitivity and specificity values, which are the quantities we have used in our benchmark.

We decided to benchmark DIVE against MGEfinder since as Durrant et al. show in their work, MGEfinder outperforms the state of the art. As a result, we consider that if we can

show that DIVE outperforms MGEfinder, then it has to outperform the tools considered in their benchmark.

We also note that virsorter is a tool for the detection of viral signals in microbial genomic data, whereas DIVE is designed to detect diversity-generating elements and mobile genetic elements in general. We think that, although there is some overlap, DIVE was designed to be a much more general tool and might not be an appropriate comparison. We believe that MGEfinder is the closest tool available.

### 9. The analysis was run using k-meres of size 27. Did you investigate the differences in the results with the k-meres size?

This is another interesting point that we just didn’t have room to discuss at more length in our paper. The rationale behind this choice follows that of some of the other parameters that we discussed above: striking a balance between statistical power and memory requirements.

On the one hand, very small k-mer lengths will result in little sequence specificity. For example, setting *k=3* will result in k-mers that are not specific to any loci in the genome since any trinucleotide is abundant in the genome and there is little specificity. Thus, we set to use large k-mers to avoid this problem.

On the other hand, increasing the k-mer size results in an exponential increase in the number of anchors to be stored in the anchor dictionary, which inevitably leads to higher RMA memory requirements. Furthermore, increasing the k-mer length past a certain point could also be counterproductive. For example, CRISPR repeats are anywhere between 27-30 bp in length. Thus, if we set k=35, then it is very likely that DIVE would miss these elements since there would not be a conserved sequence of length 35 for which we could observe the target variability (spacer in this case). Similarly, the termini of MGE are conserved to a certain extent, thus increasing *k* past a certain extent could be problematic for the same reason.

Overall, we found that setting *k*=27 results in a good balance between all these considerations, resulting in an algorithm that scales up to millions of reads and that, at the same time, has good specificity and discovery power.

### 10. In the supplementary material Table S1, do the anchors correspond to the assembled anchors or anchors used for assembly?

The anchors in Table S1 include all significant anchors. As outlined above, the anchors used during the assembly step are the ones that show significance in a single direction, since we expect that MGE termini will show variability on the insertion side, but not in the opposite direction (inwards of the element).

### 11. In the supplementary Table S1, all the anchors only have one annotation. Is that possible that a single anchor hits different databases? And how do the authors manage when there are two exactly the same hits in different databases?

It is indeed possible that one anchor hits more than one database, but as explained in the Methods section *Annotation of DIVE’s output*, we pick the hit that produces the lowest BLAST e-value. If the sequence hit two different databases, we still assign the anchor the label that produced the lowest e-value.

### 12. In ﬁgure 1A, the anchors are represented as the internal part of the transposable element. It is not clear if the anchor is necessarily inside the MGE or can be outside its boundary. According to the methods, it seems that the anchors can be the outer boundary of the MGE if this is a chromosomal region with a high recombination rate (cf Figure here).

Figure 1A shows the case where an active TE inserts itself in different loci across a set of genomes (see Figure S2b). Although, the k-mer on the boundary of the element will show some target diversity (case where the element is not there vs case where it is), the effect size, in this case, will be small. However, the termini of the element shown in red (5’ end) and green (3’ end) will show larger target variability due to the different insertion sites. If this was an ancestral element (see Figure S2a), then we would not expect any variability at all on the boundaries, and we will also expect large variability on the MGE termini.

Thus, when it comes to MGE insertions, we expect the anchor to be the termini of the element since this is the part of the element that will appear in different genetic contexts, generating large target diversity upstream for the 5’ end of the element and downstream for the 3’ end of the element. In general, we only expect a significant anchor to be part of the genome (excluding inserted MGEs) when it comes to a diversity-generating element such as CRISPR. In this case, the anchor is an integral part of the genome, and the variability is observed due to surrounding changes in sequence composition at the same locus.

### 13. Fig 1: The legend goes from Fig 1a to Fig1f while there is only a-e in the ﬁgure. It seems that d-e legend is about the same subﬁgure.

The text in f was the result of a panel that was removed from an earlier version of the figure. This has been fixed in the updated version of the paper.

### 14. Precise that it works on reads in the abstract.

This has been clarified in the abstract.

### 15. Fig 2: what is purple: conjugation gene?

We believe that the reviewer is referring to the color bar under each ICE element in panel d. The color bar shows the normalized anchor coverage and shows the concentration of anchors around the integration hotspots, which are the part of ICEs that confer different phenotypes to the host genome. This shows how DIVE can be used to study within-element variability as well as discover *de novo* integration hotspots, which are critical to understanding the role that ICE play.

### 16. We tried to use DIVE on our own dataset to ﬁnd MGE. We used 34 E. coli strains with 500k reads of 150 bp for each strain. We ran DIVE with the conﬁguration set to 27 k-mers. Is this dataset sufﬁcient to run DIVE and have robust results? Is it possible to have an efﬁciency benchmark on the quality of the results with the amount of data used?

Since DIVE controls for coverage when doing statistical analysis, low coverage might lead to little statistical power, but the method should still control for type I error in any case. So if the reviewer is using robustness in the sense of controlling the type I error, then that should be the case.

As with any statistical method, increasing the coverage will generally lead to better performance, as shown in our simulations. The benchmark the reviewer suggests is already available in our simulations where we produce ROC curves for various coverages in over 100 different scenarios varying the mutation and indel rate, as well as the copy number and activity level of the MGE (see Figure 1.e, Figures S3-S6).

In this particular case, 500k 150 bp reads from *E. coli* will result in a ~15x coverage, which is more on the lower end. In this regime, we would expect good sensitivity for termini of “ancient” MGEs with copy numbers larger than 2, as Figure S5 shows. However, we do not expect a lot of sensitivity for “active” elements with a single copy per genome, as Figure S6 shows.

### 17. In the results ﬁle, we have 1314 assembled anchors. We then annotated those results using TnCentral and ICEberg as annotation ﬁles. The annotation process of DIVE is not clear. Which part of the result is a blast against the database? The assembled anchors? The different anchors? Or the target? Can the authors give more information for this step in the manuscript or in the readme?

We are very excited that the reviewer was able to successfully run our tool and obtain meaningful results.

For each anchor cluster, we choose a representative anchor (RAs) for each direction. The upstream RA is assigned to the anchor that shows the largest effect size in the upstream direction. Similarly, the downstream RA is assigned to the anchor that shows the largest effect size in the downstream direction. These are the two anchors that are annotated.

This was explained in the *Supplementary Information* section *Algorithmic details*. However, we realize that it might not have been very clear. As a result, to address this question we have clarified that the annotation is performed on the upstream and downstream RA for each anchor cluster in the *Annotation of DIVE’s output* in *Methods*, as well as on the README file available on DIVE’s Github [repository](https://github.com/jordiabante/biodive/blob/master/README.md).

### 18. The annotation results in the ﬁle “*_anchors_annot.txt.gz” does not have a lot of information. There is only one hit (not the number of hits in the database) and just the evalue (no identity or coverage). Can the authors add more information to this output?

The annotation currently includes the best hit, which is the one that minimizes the e-value. The information includes the database, the entry within the database, and the e-value. If one desires to look further into the details of the annotation, the intermediate XML files that are produced during the annotation are available if one desires to dig deeper into the alignment statistics as the Github repository explains. However, in future updates of the tool, we can further expand the information that is automatically included in the file mentioned by the reviewer.

### 19. By Blasting the assembled anchors on ICE_seq_all.fas (ICEBerg database) and transposons.fna from TnCentral, we found several hits. TnCentral hits with 92 different sequences. ICEBerg with 33 sequences. 20 sequences were found by both databases (including 10 with exactly the same score/identity/coverage). Can the authors explain how the annotation process can determine the type of MGE?

What the reviewer discusses is the expected behavior when there is an overlap between databases. As previously discussed, the annotation of any given anchor is chosen so that it minimizes the corresponding e-value. This quantity is largely a function of the size of the database, with larger databases resulting in larger e-values generally since there are higher chances of producing hits just by chance. Thus, the annotation will tend to prioritize hits to smaller databases. However, if that is not the desired behavior, then we would suggest ensuring there is no overlap between databases.

This is not a limitation of DIVE, but an inherent result of blasting k-mers to multiple databases. As discussed above, the reviewer can look at the intermediate XML files that are produced, which contain all the necessary information. The information presented in the

*_anchors_annot.txt.gz* in the first version of DIVE is just meant to be a summary, but we will consider adding further information to it in future updates.

### 20. We then blasted all the assembled anchors on a complete genome database of E. coli, to look if we can ﬁnd those anchors in complete genomes to ﬁnd MGE. A lot of anchors were found in ncRNA (as reported in the manuscript) but in a bigger proportion. Is this linked to the small dataset used for testing?

There are many potential explanations for the observed behavior. It is a little difficult to judge what might be the underlying reason without having access to the results, but here we share our thoughts in case this can be helpful.

For low coverage settings, only anchors with very large effect sizes will have enough statistical power to be called significant. Note this is a general property of any statistical method, and indeed a desirable one. This means that in the other cases, there is just too much variability to say anything for sure.

We imagine that the anchors mapping to ncRNA are probably mapping to a large number of loci in the genome (perhaps conserved sequences across ncRNA?), conferring them with high statistical power. Perhaps, increasing the coverage would lead to the discovery of other k-mers with weaker effect sizes.

We are also not sure what FDR threshold the reviewer is using, which would also condition the distribution of positives. For example, picking a cutoff of 0.01 will surely prioritize very strong effect sizes. There is also a possibility that that is the real distribution of true positives in the available data. But, again, this is hard to judge without having access to the actual results.

## Reviewer 2

### 1. Abante et al present a new method for detecting mobile genetic elements, using a reference free approach. Overall I think this is a worthy goal and deserving of a new tool. I think the authors present a sensible algorithm and a nice approach for tackling this problem. However, my biggest issue with the manuscript was that I found it very difﬁcult to follow along, especially trying to pair the ﬁgures with their relevant sections in the text. I would recommend that the authors signiﬁcantly revise their manuscript for clarity, and consider extracting sections of the supplementary text and placing it into the main text to improve readability.

We have modified the text so it is easier to pair the figures with their text counterpart. We have also worked on the figure legends to include more detail to help the reader better understand the figure. Lastly, we have also included more detail in certain sections following the reviewer’s 1 suggestion (see above).

### 2. For example, for ﬁgure 1, each panel (aside from e) appears to visually describe how the algorithm works. However in the text, Figure 1 is just referred to in its entirety, and the reader is forced to try and read the ﬁgure legend to work out which panel of the ﬁgure refers to which description in the text. This is made extra difﬁcult by the lack of detail in the ﬁgure legend – eg for ﬁg 1a, how is the reference to sequencing adapters relevant to this ﬁgure? And for Fig 1b, what is the “logic” referred to with a FastQ

***ﬁle? What are each of the coloured boxes – are all boxes with a gradient an anchor? Or are just the green boxes anchors, and the other colours targets, regardless of gradient. JS presumably means jaccard similarity? This isn’t explained until Fig 1c. How does the depiction in panel 1d correspond with steps of the algorithm?***

We have reorganized Figure 1 and modified the references to the figure so that the specific panel is referenced. We have also clarified within the figure itself that JS stands for Jaccard similarity as the reviewer points out. Lastly, we have also improved the figure legend substantially, adding much more detail than in the previous version, and addressing the questions raised by the reviewer.

### 3. Figure 2 is also difﬁcult to follow along with the main text. Are all the panels in the ﬁgure referenced in the main text? I was confused about how panel 2c ﬁt in with what is described in the main text – there doesn’t appear to be much mention of Rotavirus? It seems like this information in the supplementary text? Generally, the main text should contain all the information required to understand the main ﬁgures, and readers shouldn’t have to dig through the supplementary to simply understand what is in the main ﬁgures. Also, for ﬁgure 2b, is there any reason why are you showing target diversity compared to environments that the E. coli were found in, rather than genetic background of the E. coli (eg sequence type?) I’m not sure what the relevance of the higher diversity in certain environments is, if there is any at all?

The specific panels were not referenced in the main text, but this has been addressed in the updated version of the manuscript.

Panel 2c shows a putative CRISPR repeat discovered *de novo* in human gut metagenomic samples of subjects with a *Rotavirus* infection. The panel shows the context in which the repeat was found upon blasting against the NCBI nucleotide database. We have consolidated how we refer to this dataset across the main and supplementary text, as well as in the supplementary information, to avoid any confusion. As mentioned above, we are very much limited by the word count and hence we need to refer the reader to the Supplementary Information in certain parts of the manuscript for further information.

Nevertheless, we have added a little bit more detail to this part of the text so that the main idea is properly conveyed.

Panel 2b shows the diversity observed for a given CRISPR repeat that was found de novo by DIVE. By construction, this implies that the repeat is followed (and preceded) by a highly diverse set of target sequences in the isolation sources on the x-axis. In fact, this implies that the diversity observed is much higher than expected under a null model that incorporates the biological and technical variability in the data. Furthermore, the target variability of this specific repeat is shown to be a function of the isolation source since, during our association analysis, we find that the diversity in three environments is significantly larger than in the rest (after correction for multiple hypothesis). Although we do not have an mechanistic explanation for this observation, we consider this result is

interesting per se given that this is not the case for most other CRISPR repeats (we only found 5 such instances), let alone the majority of k-mers.

### 4. For the M. tuberculosis analysis, why were there nine clusters that all matched the same IS, IS6110? Were the contigs longer than the IS itself, containing other sequence data? I would have assumed that all copies of IS6110 in the genome would be almost identical, and so I’m unsure why they are being clustered out into so many different clusters?

Putative MGE termini were obtained for each sample by picking anchors showing variability in only one direction since that fits the expected behavior in this case. These termini were then used as seeds in a seed-based assembly step where we used SSAKE iteratively to assemble a longer sequence from these anchors. This process resulted in a set of contigs per sample, which we filtered first searching for transposon-associated domains and we then clustered to reduce the cluster redundancy as much as possible. However, it is still possible that, due to partial reconstruction of the element, some contigs overlapping the same element would have a small overlap between them, resulting in them not being clustered together. Nevertheless, upon aligning them we can see that they indeed represent different parts of the same element and thus we can see multiple clusters being associated with *IS6110*.

### 5. Species names in abstract/main text should be fully typed out on ﬁrst use, eg Escherichia coli not E. coli. Also, M. tuberculosis should be lowercase ‘t’ as it is species name.

This has been fixed in the main text, figures, and supplementary information.

### 6. There are a couple of unfamiliar acronyms. RA is uncommon, can that just be spelt out clearly? Also the acronyms for DGMs and DGRs seem unnecessary, they are only used once or twice and are not standard in the ﬁeld?

Representative anchor (RA) is spelled out in the main text: *“Lastly, anchors are clustered to reduce redundancies in the output, and a representative anchor (RA) maximizing the effect size (...)”*. We consider that RA is used enough times to keep it as an acronym.

Regarding the acronym DGM, it is used 5 times just along the main text, and, in turn, we consider that it is fine keeping it as such. As for DGR, we have removed it from the main text since it is indeed only used once and the acronym was unnecessary as the reviewer points out.

**First round of review**

**Reviewer 1**

I am happy with the changes the authors have made to the manuscript in response to my comments.

**Reviewer 2**

We felt the manuscript has much improved and that the authors have adequately addressed our comments.

Notably, lots of improvements concerning the readability of the paper, which makes clearer the goal of the method.
Still a couple remarks :
- Unanswered point : In figure 2d : Genes are colored based on their annotation (integrase, antibiotic resistance). Yet, some genes are colored in purple/mauve but the reader don't know to what it corresponds. On this figure, why ICEVchBan8 has no annotations at all ? Still on this figure, in a, where do we see that TnXAj417 and TN3434 have the largest effect size? Should the author adds a second dimension to the bar plot ?
- Since the authors are working with Vibrio cholerae, we wondered why Vibrio Cholerae Repeats (corresponding to attC sites of the super integrons of vibrio cholerae) are not picked up by the method. VCR are mostly conserved region flanked by variable genes, and there are hundreds of them (up to 200 in some strains, but most avec >100). Could the author discuss why they are not detected (maybe repeats are too degenerated?) and whether this method could discover this type of mobile genetic elements which are a major driver of antibiotic resistance spread.
- adding the SSAKE + HMMER part to the package, even turned off by default, or proposing different options for it (e.g. option to specify which HMM databases to use for the annotation), would be greatly appreciated by the larger community of biologists which are not proficient in installing and running various piece of software.

**Authors’ response to reviewers**

We would also like to thank both reviewers for their insightful comments and suggestions which have improved both the quality and the presentation of our work. We really appreciate it.

Here are our answers to the remaining questions from reviewer 2:

1. In ﬁgure 2d: Genes are colored based on their annotation (integrase, antibiotic resistance). Yet, some genes are colored in purple/mauve, but the reader doesn't know to what it corresponds.
We have added the mauve elements, which represent components of type IV secretion systems (T4SS), to the legend as well as the caption for completeness.

2. On this ﬁgure, why does ICEVchBan8 have no annotations at all?
Unfortunately, the database we used (ICEberg2) did not have annotations for ICEVchBan8 and so we couldn’t add these.

3. Still on this ﬁgure, in a, where do we see that TnXAj417 and TN3434 have the largest effect size? Should the author add a second dimension to the bar plot?
The effect size of TnXAj417 and TN3434 can be seen in Table S4. We notice, however, that this was not explicitly mentioned in the figure caption, which we have now added for clarity.

4. Since the authors are working with Vibrio cholerae, we wondered why Vibrio Cholerae Repeats (corresponding to attC sites of the super integrons of vibrio cholerae) are not picked up by the method. VCR are mostly conserved region ﬂanked by variable genes, and there are hundreds of them (up to 200 in some strains, but most avec >100). Could the author discuss why they are not detected (maybe repeats are too degenerated?) and whether this method could discover this type of mobile genetic elements which are a major driver of antibiotic resistance spread.
Given the characteristics of VCR described by the reviewer we are convinced that DIVE could identify these repeats. This follows from the fact that VCRs consist of a conserved region flanked by variable sequences, which is precisely a signal for which DIVE is statistically powerful as shown in our simulations and real data analysis. In our work we focused on SXT ICE elements in V. cholerae given their important role in antibiotic resistance. However, we believe that some of the unannotated representative anchors identified by DIVE in V. cholerae probably constitute VCRs. Furthermore, we also believe that the proposed de novo MGE discovery approach, or a slightly optimized version (see question 5), could potentially reconstruct the entire super integron sequence.

5. Adding the SSAKE + HMMER part to the package, even turned off by default, or proposing different options for it (e.g. option to specify which HMM databases to use for the annotation), would be greatly appreciated by the larger community of biologists which are not proﬁcient in installing and running various piece of software.
We completely agree with the reviewer that this would be a nice addition to DIVE. Both SSAKE and HMMER, as well as DIVE, are user-friendly tools that can be easily installed and run following the steps described in our work with basic programming skills. Nevertheless, other members of the Salzman lab are currently developing custom tools that can be run on the output generated by DIVE to perform downstream analysis. These tools will address the shortcoming of SSAKE, for example, which is not optimized for this application since one cannot leverage all the information in DIVE’s output (as discussed in the previous revision round). Thus, in future iterations we will consider adding these tools to the biodive package as well as creating a reference-free suite of computational tools where all these methods (and others) would be available right off the bat making this work accessible to a broader community.
